# A Comparison of Breastfeeding Exclusivity and Duration Rates Between Immediate Postpartum Levonorgestrel Versus Etonogestrel Implant Users: A Prospective Cohort Study

**DOI:** 10.1089/bfm.2018.0165

**Published:** 2019-01-11

**Authors:** Jamie W. Krashin, Clara Lemani, Jerome Nkambule, George Talama, Lameck Chinula, Valerie L. Flax, Alison M. Stuebe, Jennifer H. Tang

**Affiliations:** ^1^ Department of Obstetrics and Gynecology, University of North Carolina School of Medicine, Chapel Hill, North Carolina.; ^2^ Department of Obstetrics and Gynecology, University of New Mexico, Albuquerque, New Mexico.; ^3^ UNC Project-Malawi, Lilongwe, Malawi.; ^4^ Kasungu District Hospital, Kasungu, Malawi.; ^5^ Kamuzu Central Hospital, Lilongwe, Malawi.; ^6^ Partners in Health, Neno, Malawi.; ^7^ RTI International, Research Triangle Park, North Carolina.; ^8^ Department of Maternal and Child Health, Gillings School of Global Public Health, Chapel Hill, North Carolina.

**Keywords:** breastfeeding, lactation, contraceptive implant, immediate postpartum, progestin, Malawi

## Abstract

***Objective:*** This study compares breastfeeding outcomes after immediate postpartum initiation of single-rod etonogestrel (ENG) versus two-rod levonorgestrel (LNG) contraceptive implants. Outcomes assessed include the following: (1) breastfeeding continuation through 24 months after delivery and (2) exclusive breastfeeding until 6 months after delivery, at Kasungu District Hospital, Malawi.

***Methods:*** We used Kaplan–Meier survival analysis to compare breastfeeding continuation through 24 months and exclusive breastfeeding through 6 months after delivery for ENG versus LNG implant users. We described infant feeding practices up to 6 months after delivery.

***Results:*** We analyzed 140 women: 28 (20%) ENG and 112 (80%) LNG impalnt users. Eighty-seven percent (*n* = 122) of women completed the 24-month study visit. Twenty-four months breastfeeding continuation proportions were 54.2% (95% confidence interval [CI] = 32.7–71.4) and 74.7% (95% CI = 64.9–82.2) for ENG and LNG implant users, respectively (*p* = 0.10). Breastfeeding continuation was high in both groups at 21 months: 100% and 93.2% (95% CI = 86.2–96.7) for ENG and LNG implant users, respectively (*p* = 0.18). Seventy-one percent (20/28, 95% CI = 51.0–84.6) of ENG and 72% (78/108, 95% CI = 62.4–79.7) of LNG implant users exclusively breastfed their infants until 6 months postpartum (*p* = 0.89).

***Conclusions:*** Continuation of breastfeeding until 24 months and exclusive breastfeeding until 6 months were high among users of both types of progestin implant initiated immediately postpartum and similar to proportions among the general population of postpartum women in the Central region of Malawi.

## Introduction

Breastfeeding and access to contraception are important considerations for postpartum women. The World Health Organization (WHO) recommends that women exclusively breastfeed for 6 months after delivery and then continue breastfeeding, along with appropriate complementary foods, until their child is 2 years or older for both the maternal and infant benefits.^1,2^ For women who are <6 months postpartum and have not resumed menses, exclusive breastfeeding provides an effective, but temporary, method of contraception.^3^ Access to long-acting reversible contraception during the immediate postpartum period (<48 hours after placental delivery) enables interested women to control their childbearing beyond the first 6 months postpartum, without having to return to a health facility for family planning. Given that transportation costs, inadequate clinic implant stocks, and the need to return for a separate insertion visit limit women's access to desired postpartum implants, immediate initiation has the potential to help women to avoid short interpregnancy intervals, which may be linked to early breastfeeding cessation and adverse subsequent pregnancy outcomes.^4–9^

Two progestin contraceptive implants, the 3-year single-rod etonogestrel (ENG) single-rod implant and the 5-year two-rod levonorgestrel (LNG) two-rod subdermal implant, offer highly effective reversible contraception for 3 and 5 years, respectively.^10^ Because of the role of progesterone withdrawal in lactogenesis II, theoretical concerns exist about the potential effect of initiating progestin contraception immediately postpartum on breastfeeding performance.^11^ However, the clinical implications of this theoretical risk to breastfeeding are uncertain. WHO recommends that breastfeeding women can generally use both these progestin implants before 6 weeks postpartum based on very low-quality evidence.^3^ This recommendation is based on clinical trials and observational studies that do not suggest an increased risk of suboptimal breastfeeding practices comparing (1) one type of implant initiated before 6 weeks with initiating the same type of implant at least 6 weeks postpartum, (2) one type of implant with a nonhormonal method when both are initiated before 6 weeks postpartum, or (3) indirect evidence from one type of implant with a different hormonal method when both are initiated before 6 weeks postpartum.^3,12^

To date, no studies have compared immediate postpartum initiation of ENG with LNG implants on breastfeeding outcomes. No studies examine immediate postpartum initiation of either progestin implant on breastfeeding practices outside the United States or Brazil. Finally, no immediate postpartum progestin implant studies follow women for the 24 months of breastfeeding recommended by WHO. A recent immediate postpartum implant implementation project at a district hospital in Kasungu, Malawi provided the opportunity to address these gaps in the literature. Our objectives were therefore to compare exclusive breastfeeding practices until 6 months and continued breastfeeding until 24 months in ENG and LNG implant users.

## Materials and Methods

This was a prospective cohort study of women receiving an immediate postpartum contraceptive implant at Kasungu District Hospital in Malawi. Kasungu District Hospital is a government-funded hospital for this rural district in central Malawi. Women were recruited between September 2014 and March 2015, during which time the hospital was implementing an immediate postpartum ENG and LNG implant insertion program. The University of North Carolina at Chapel Hill Institutional Review Board and the Malawi National Health Services Research Committee approved both the implementation program and this study.

Eligibility criteria included age 18 years or older, initiating a contraceptive implant within 48 hours of delivery, and willingness to return for visits every 3 months for 24 months. Women could choose either the LNG or ENG implant. Study nurses recruited a convenience sample of all interested eligible women on the postpartum ward during the 6.5-month recruitment period. After undergoing informed consent, participants completed a research assistant-administered baseline survey in Chichewa. They attended study visits every 3 months, at which they completed follow-up surveys and had implant continuation confirmed by arm palpation. Transportation costs were reimbursed at each study visit. Participants were followed for 2 years after delivery. We excluded participants from the analysis who had an intrauterine fetal or infant demise before first follow-up due.

Exposure and potential confounding variables were measured before hospital discharge by examination and survey. The exposure—type of implant—was recorded by study nurses in the hospital. Research assistants asked participants about demographic characteristics, reproductive history, and breastfeeding expectations in the baseline survey.

The outcome variables were self-reported breastfeeding continuation until 24 months and exclusive breastfeeding until 6 months. Participants were asked if they had completely stopped breastfeeding or expressing milk to measure breastfeeding continuation at each follow-up visit through 24 months. The infant's age in months at which they stopped was recorded if applicable. They were also asked if their baby had been fed anything other than the participant's own milk since birth, and if so, with what and at what age in months. We asked women for the reason(s) they supplemented before 6 months or stopped breastfeeding through 24 months. We assessed infant feeding using questions adapted from the Infant Feeding Practices Study II neonatal and postnatal questionnaires.^13^ If a participant's answer at a later follow-up visit conflicted with an earlier answer, we used the earlier follow-up visit answer to limit recall bias.

We conducted descriptive, bivariate, and survival analyses. We used Pearson's chi-squared or Fisher's exact tests, as appropriate, to compare baseline characteristics by implant type. We then calculated breastfeeding continuation using Kaplan–Meier estimator of survival analysis. Participants were considered lost to follow-up after the last completed survey. They were censored at the visit before loss to follow-up or infant death. We used the log-rank test of equality between the two types of progestin implants for breastfeeding continuation until 24 months and planned to adjust for confounding using Cox proportional hazards modeling. Finally, we described timing and type of supplementation, as defined by initiation of nonbreast milk fluids and foods, of infants who were not exclusively breastfed until 6 months. Data were double entered into Microsoft Access and analyzed using Stata 14.0.

## Results

**FIG. 1.**
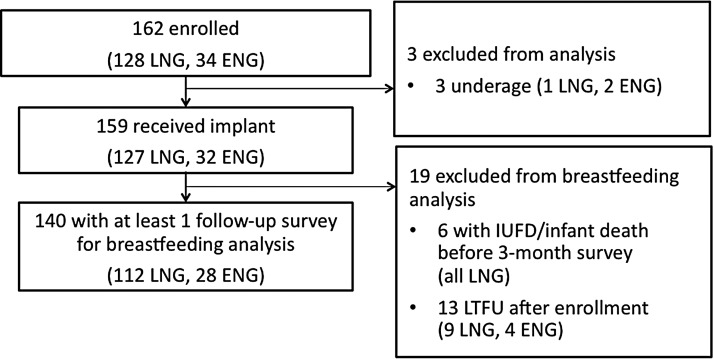
Flow diagram. ENG, single-rod etonogestrel implant users; IUFD, intrauterine fetal demise; LNG, two-rod levonorgestrel implant user(s); LTFU, lost to follow-up.

We enrolled 162 women who had an immediate postpartum implant placed from September 2014 to March 2015, which included 100% of women who received an immediate postpartum implant during that time period (Fig. 1). We excluded three women who were found to be underage after enrollment, six women with an intrauterine fetal demise or infant death before the first follow-up survey at 3 months—all of which were unrelated to malnutrition, and 13 women lost to follow-up before 3 months postdelivery. Our final sample for analysis was 140 participants: 28 (20%) ENG and 112 (80%) LNG implant users.

Table 1 shows baseline participant characteristics. ENG implant users were more often <25 years (21/28 [75%]) compared with LNG implant users (50/112 [45%]) (*p* < 0.01) and had fewer children: 46% (13/28) of ENG implant users had only the child she just delivered compared with 9% (10/112) of LNG implant users (*p* < 0.01). ENG implant users were also more likely to want more children in the future: 93% (26/28) compared with 61% (61/111) LNG implant users (*p* < 0.01). ENG implant users (50% [14/28]) were less likely to have ever used a family planning method than LNG implant users (87% [78/112]) (*p* < 0.01). ENG implant users were slightly more likely to report planning to introduce fluids and foods before 6 months: 11% (3/28) versus 1% (1/112) for sugar water or juice (*p* = 0.02) and 7% (2/28) versus 0% (0/111) for solids (*p* < 0.01). ENG implant users trended toward higher educational attainment and desiring this pregnancy at the time of conception compared with LNG implant users, although these differences were not statistically significant.

**Table 1.** Baseline Characteristics Before Hospital Discharge of Women Who Received an Immediate Postpartum Implant in Kasungu, Malawi

		*Type of implant*		
*Characteristic*	*Total,* N* = 140,* n *(%)*	LNG, n = 112 (80%)	ENG, n = 28 (20%)	p
Age, years				<0.01
18–25	71 (51)	50 (45)	21 (75)	
26–34	56 (40)	51 (46)	5 (18)	
35–42	13 (9)	11 (10)	2 (7)	
Married	136 (97)	109 (97)	27 (96)	0.80
Highest level of education				0.11
None to full primary	93 (66)	78 (70)	15 (54)	
Some secondary or higher	47 (34)	34 (30)	13 (46)	
Roof made of grass	91 (65)	69 (62)	22 (79)	0.09
HIV status				1.0
Negative	137 (98)	109 (97)	28 (100)	
Positive	2 (1)	2 (2)	0 (0)	
Do not know	1 (1)	1 (1)	0 (0)	
No. of living children				<0.01
1	23 (16)	10 (9)	13 (46)	
2–3	65 (46)	54 (48)	11 (39)	
4–5	33 (24)	31 (28)	2 (7)	
6–8	19 (14)	17 (15)	2 (7)	
Desires more children (*n* = 139; 111 LNG)	94 (68)	68 (61)	26 (93)	<0.01
This pregnancy was desired at time of conception	65 (46)	48 (43)	17 (61)	0.09
Ever used a family planning method	101 (72)	87 (78)	14 (50)	<0.01
Ever used an implant (*n* = 99; 85 LNG, 14 ENG)	7 (7)	6 (7)	1 (7)	1.0
Have friends who have used the implant (*n* = 139; 111 LNG)	100 (72)	81 (73)	19 (68)	0.59
Partner aware that she is using implant (*n* = 137; 109 LNG)	100 (73)	81 (74)	19 (68)	0.49
Mode of delivery (*n* = 136)				1.0
Vaginal	128 (94)	102 (94)	26 (96)	
Cesarean	8 (6)	7 (6)	1 (4)	
Within how many minutes did you first attempt to start breastfeeding after delivery (*n* = 139)				0.76
Within 30 minutes	88 (63)	70 (63)	18 (64)	
Within 30–60 minutes	44 (32)	36 (32)	8 (29)	
After 60 minutes	7 (5)	5 (5)	2 (7)	
When participant breastfed infant (*n* = 139)				0.12
When the infant seems hungry	128 (92)	104 (94)	24 (86)	
On a schedule	1 (1)	0 (0)	1 (4)	
Sometimes when hungry, sometimes on a schedule	10 (7)	7 (6)	3 (11)	
Infant age at which participant planned to supplement				
Plain water				0.18
1–3 months	6 (4)	4 (4)	2 (7)	
4–5 months	7 (5)	6 (5)	1 (4)	
6–8 months	126 (90)	102 (91)	24 (86)	
Do not know	1 (1)	0 (0)	1 (4)	
Sugar water/juice				0.02
3–4 months	4 (3)	1 (1)	3 (11)	
6–12 months	126 (90)	102 (91)	24 (86)	
Do not know	10 (7)	9 (8)	1 (4)	
Coffee/tea				0.23
3 months	1 (1)	0 (0)	1 (4)	
6–12 months	132 (94)	106 (95)	26 (93)	
Do not know	6 (4)	5 (4)	1 (4)	
Do not plan to introduce	1 (1)	1 (1)	0 (0)	
Infant formula				0.21
6–12 months	126 (90)	99 (88)	27 (96)	
Do not know	14 (10)	13 (12)	1 (4)	
Yogurt				0.54
4–5 months	2 (1)	1 (1)	1 (4)	
6–12 months	125 (89)	101 (90)	24 (65)	
Do not know	13 (9)	10 (9)	3 (11)	
Other liquids				0.03
3–5 months	2 (1)	0 (0)	2 (7)	
6–12 months	134 (96)	109 (97)	25 (89)	
Do not know	3 (2)	2 (2)	1 (4)	
Do not plan to introduce	1 (1)	1 (1)	0 (0)	
Solids foods (porridge, etc.) (*n* = 139)				<0.01
3–5 months	2 (1)	0 (0)	2 (7)	
6–12 months	137 (99)	112 (100)	25 (93)	
Animal milk				0.17
6–11 months	125 (89)	98 (88)	27 (96)	
12–15 months	15 (11)	14 (13)	1 (4)	
Infant age at which participant anticipated completely stopping breastfeeding (*n* = 139)				0.31
18–24 months	87 (63)	72 (64)	15 (56)	
25–36 months	47 (34)	37 (33)	10 (37)	
Do not know	5 (4)	3 (3)	2 (7)	

ENG, single-rod etonogestrel implant users; LNG, two-rod levonorgestrel implant users.

Other characteristics of ENG and LNG implant users were similar. Almost all participants were married, HIV negative, and had a vaginal delivery. The majority of participants in both groups had less than a secondary education and a grass roof on their homes, indicating lower socioeconomic status. Few participants had ever used an implant themselves, but most had friend who had used the implant. Most reported that their partners were aware they were using the implant. Almost all had started breastfeeding within an hour of delivery, and most were breastfeeding when the infant seemed hungry. Almost two-thirds of participants planned to stop breastfeeding between 18 and 24 months.

**FIG. 2.**
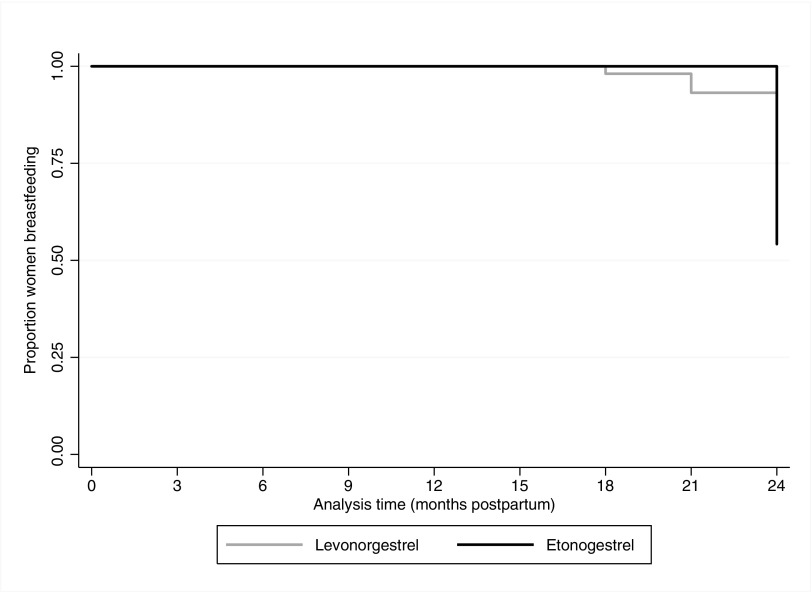
Kaplan–Meier survival curves: breastfeeding continuation through 24 months postpartum.

Figure 2 shows the Kaplan–Meier survival curves for breastfeeding continuation by progestin type. Eighty-seven percent (122/140) of women completed a 24-month study visit: 85.7% (24/28) of ENG and 87.5% (98/112) of LNG implant users. Breastfeeding continuation proportions until 24 months were 54.2% (95% confidence interval [CI] = 32.7–71.4) and 74.7% (95% CI = 64.9–82.2) for ENG and LNG implant users, respectively (*p* = 0.10). Almost all women who were not breastfeeding at 24 months reported discontinuing at the 24-month follow-up visit. Breastfeeding continuation at the 21-month visit was 100% and 93.2% (95% CI = 86.2–96.7) for ENG and LNG implant users, respectively (*p* = 0.18). All women who discontinued breastfeeding reported not having enough milk to satisfy their babies at the visit they stopped breastfeeding.

**FIG. 3.**
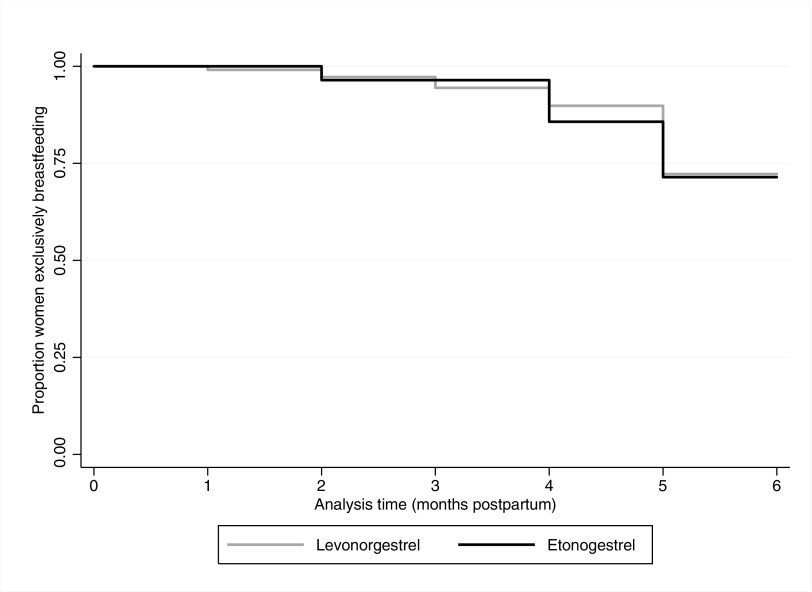
Kaplan–Meier survival curves: exclusive breastfeeding through 6 months postpartum.

Table 2 describes reported introduction of nonbreast milk fluids or foods to the infant before 6 months postpartum by both types of implant users. Among women with follow-up data through 6 months, 71% (20/28) of ENG and 72% (78/108) of LNG implant users exclusively breastfed their infants until 6 months postpartum (Fig. 3). The majority of women introducing fluids and foods early did so starting at 5 months in both groups. Only seven women introduced fluids and foods before 4 months: 4% (1/28) ENG implant users and 6% (6/108) LNG implant users. Most women gave their infants plain water and/or other liquids, especially among those who started before the infant was 4 months. All women who supplemented before 6 months reported not having enough milk to satisfy their babies. We were unable to perform planned Cox proportional hazard modeling for our two outcomes because of low enrollment of ENG implant users.

**Table 2.** Supplementation Before 6 Months Among Women Who Received an Immediate Postpartum Implant in Kasungu, Malawi

*Infant age at supplementation (months)*	*LNG,* n *(%)*	*ENG,* n *(%)*	*Supplement*	*Cumulative total women supplementing*
1	1 (1)	—	Coffee/tea	1
2	2 (2)	1 (4)	Plain water	4
3	1 (1)	—	Plain water	7
	1 (1)		Solids	
	1 (1)		Sugar water/juice	
4	1 (1)	2 (7)	Plain water	15
	3 (2)	1 (4)	Plain water and solids	
	1 (1)	—	Coffee and solids	
5	8 (7)	3 (7)	Plain water	38
	—	1 (1)	Plain water and animal milk	
	1 (1)	—	Plain water and sugar water/juice	
	4 (4)	—	Solids	
	4 (4)	—	Plain water and solids	
	2 (2)	—	Plain water, sugar water/juice and solids	
Total	30/108^a^ (28%)	8/28 (29%)		

^a^ Four LNG users were removed from analysis after 3 months: three were lost to follow-up and one was removed after her infant died from a cause unrelated to malnutrition. All reported breastfeeding exclusively at 3 months postpartum.

One woman who was removed from analysis after discovering that she was underage and likely had failure of lactogenesis Stage II. She had an ENG implant placed and later reported not being able to produce milk for this infant and a prior infant. If she were included in the analysis, then the risk of lactation failure would be 0.7% (95% CI = 0.02–3.9) for either LNG or ENG implant users and 3.4% (95% CI = 0.1–17.8) for ENG implant-only users.

## Discussion

Most women initiating immediate postpartum implants in a rural district hospital in central Malawi successfully breastfed their infants as measured by 24-month continuation and exclusive breastfeeding until 6 months postpartum. This success did not vary by type of progestin implant. Participants in our study continued breastfeeding at similar proportions as those from a representative sample of households surveyed in the Central Region of Malawi from 2015 to 2016.^14^ From 2015 to 2016, 72.8% (95% CI = 66.2–78.5) of infants 20–23 months of age in the central region of Malawi were breastfed.^14^ This is lower than the continuation proportions for participants in our study at 21 months and similar to our participants at 24 months. Likewise, the proportion of infants exclusively breastfeeding from 0 to 5 months of age in the central region in 2015–2016 was similar to our participants: 62.9% (95% CI = 57.2–68.2),^14^ compared with >70% in our study. Our sample of women who delivered in a hospital, wanted contraceptive implants, and agreed to be in a study with quarterly visits might differ from women in a national household survey, thus contributing to slightly higher breastfeeding among infants 20–23 months old.

A large majority of both ENG and LNG implant users breastfed through 21 months, and more than half in each group reported continued breastfeeding at their 24-month follow-up visit. Our findings align with those of other studies examining the impact of immediate postpartum progestin implant initiation on breastfeeding continuation, although we followed our participants for a longer period of time than other studies. For example, a cohort study of women in the United States who received immediate postpartum LNG implant or depot medroxyprogesterone acetate (DMPA) or progestin-only pills (POPs) found no difference in breastfeeding continuation at 6 weeks postpartum compared with women using nonhormonal contraception.^15^ A randomized controlled trial of adolescents receiving immediate compared with 6-week initiation of ENG implants found no difference in breastfeeding at 3 and 6 weeks postpartum.^16^

In our study, most women in both groups exclusively breastfed until their infants were 6 months old. Almost all women exclusively breastfed for three full months postpartum. The main other fluids or foods were plain water and clear liquids, which did not seem to differ between groups. Early introduction of fluids and foods is common in Malawi.^17^

Earlier studies of immediate progestin implant initiation have not found differences in exclusive breastfeeding until 6 months postpartum compared with nonhormonal contraception or later initiation of hormonal contraception. A nonrandomized cohort study of 319 women in the United States who received immediate postpartum LNG implant, DMPA, or POP compared with nonhormonal methods found no difference in exclusive breastfeeding at 2, 4, or 6 weeks postpartum.^15^ A randomized controlled trial of 24 women in Brazil who received an ENG implant before 48 hours postpartum compared with no contraception found no difference in exclusive breastfeeding at 6 weeks postpartum nor any difference in the volume of breast milk infants consumed on either postpartum days 0 and 29.^18^ Similarly, a pilot randomized controlled trial comparing 40 women in Brazil who received an ENG implant at 24–48 hours postpartum with women who received DMPA at 6 weeks postpartum found no difference in exclusive breastfeeding at 12 weeks postpartum.^19^ Finally, a noninferiority randomized controlled trial of 69 women in the United States comparing ENG implant insertion at 1–3 days postpartum with 4–8 weeks postpartum found no difference in exclusive breastfeeding through 6 months postpartum.^20^

We know that one participant who was excluded from the study after discovering that she was <18 years old likely had failure of lactogenesis Stage II. This participant also experienced the same lactation failure with her first child in the absence of any exposure to hormonal contraception. Although data are limited, 5–15% of women may have lactation failure.^21^ The randomized controlled trial from the United States comparing immediate with delayed ENG implant insertion among 69 women reported one participant with lactation failure, defined as failure of lactogenesis Stage II within 120 hours, in the immediate group. The calculated risk difference for lactation failure between early and later initiation was 0.03 (95% CI = −0.02 to 0.08).^20^ The literature also includes one case report of abrupt infant growth deceleration and decreased maternal milk supply after initiation of an ENG implant at postpartum day 39 in the United States.^22^ The authors of this case found an additional report of decreased breast milk production after placement of an ENG implant in the U.S. Food and Drug Administration's Adverse Event Reporting System. If a causal association between progestin implants and lactation failure does exist, the incidence is likely very low and could possibly be an issue for women who have other predisposing factors for lactation failure. Although some risk factors such as prior lactation difficulties, preterm birth, and medical comorbidities may predispose women to lactation failure, we currently do not have a means of predicting which women are at risk.^23^ However, the consequences of insufficient lactation in a setting where access to clean water to prepare infant formula is limited, such as in Malawi, can be tragic.

Our study has several limitations. Women in our convenience sample chose the LNG implant 4:1 over the ENG implant. ENG and LNG implant users were different in many characteristics that might affect breastfeeding continuation. ENG implant users were younger, had fewer children, and were less likely to be done with childbearing, were less likely to have used contraception in the past, and more frequently planned to introduce other fluids or foods before 6 months postpartum. We lacked power to determine if our effect sizes were statistically significant between the two progestin implants, and we were also unable to adjust for potential confounding variables between these two groups. Despite these limitations, we were able to document reassuring breastfeeding outcomes to 24 months with both types of progestin implants. Finally, breastfeeding continuation, supplementation, and perceived milk supply were measured by self-report and may have been subject to recall bias. However, most participants answered questions about introduction of fluids or foods and breastfeeding cessation within a few weeks of making these decisions, and we were able to use infant age to corroborate responses.

## Conclusions

This is the first study to compare immediate initiation of ENG and LNG implants on breastfeeding performance. It is also the first to prospectively measure breastfeeding continuation among implant users for up to 24 months and to include women from sub-Saharan Africa. This study adds to the reassuring data from earlier studies regarding breastfeeding practices of women who wish to access immediate postpartum implants. Further implementation of immediate postpartum implants in Malawi, with careful monitoring of breastfeeding outcomes and counseling for women, particularly those who may be at risk for insufficient lactation, is warranted.
